# Nonlinear Optical Properties of Zn(II) Porphyrin, Graphene Nanoplates, and Ferrocene Hybrid Materials

**DOI:** 10.3390/ma16155427

**Published:** 2023-08-02

**Authors:** Francesca Limosani, Francesca Tessore, Alessandra Forni, Angelo Lembo, Gabriele Di Carlo, Cecilia Albanese, Stefano Bellucci, Pietro Tagliatesta

**Affiliations:** 1 Department of Chemical Science and Technologies, University of Rome “Tor Vergata”, Via della Ricerca Scientifica 1, 00133 Rome, Italy; francesca.limosani@uniroma2.it (F.L.); angelo.lembo@uniroma2.it (A.L.); pietro.tagliatesta@uniroma2.it (P.T.); 2 Department of Chemistry, University of Milan, Via C. Golgi 19, 20133 Milan, Italy; gabriele.dicarlo@unimi.it (G.D.C.); cecilia.albanese@unimi.it (C.A.); 3 CNR-SCITEC, Istituto di Scienze e Tecnologie Chimiche “G. Natta”, Via Golgi 19, 20133 Milan, Italy; alessandra.forni@scitec.cnr.it; 4 INFN-National Laboratories of Frascati Via Enrico Fermi 54, 00044 Frascati, Italy; bellucci@lnf.infn.it

**Keywords:** porphyrins, graphene nanoplates, ferrocene, nonlinear optics, hybrid materials

## Abstract

Following some previous work by some of us on the second order nonlinear optical (NLO) properties of Zn(II) *meso*-tetraphenylporphyrin (ZnP), fullerene, and ferrocene (Fc) diads and triads, in the present research, we explore the NLO response of some new hybrids with two-dimensional graphene nanoplates (GNP) instead of a zero-dimensional fullerene moiety as the acceptor unit. The experimental data, collected by Electric Field Induced Second Harmonic generation (EFISH) technique in CH_2_Cl_2_ solution with a 1907 nm incident wavelength, combined with Coupled-Perturbed (CP) and Finite Field (FF) Density Functional Theory (DFT) calculations, show a strongly enhanced contribution of the cubic electronic term γ(−2ω; ω, ω, 0), due to the extended π-conjugation of the carbonaceous acceptor moiety.

## 1. Introduction

In the last decades, many materials such as graphene [1,2,3], fullerene [4,5,6], and quantum dots [7,8,9] have attracted attention in the scientific community for their significant nonlinear optical (NLO) properties. In parallel, a huge amount of research has been devoted to the investigation of organic and organometallic chromophores, in which nonlinearity mainly arises from the so-called push–pull architecture, involving a donor and an acceptor moiety bridged by a π-delocalized spacer. Due to their thermal and chemical stability and quite good solubility, porphyrins and their metal complexes are employed in a large variety of fields, ranging from optoelectronics [10,11], catalysis [12,13,14,15], sensing technologies [16,17,18,19,20], photovoltaics [21,22,23,24,25,26,27,28], and artificial photosynthesis [29,30,31,32]. They have also been long investigated for their second order NLO properties, starting from the pioneering work by Therien [33,34,35] to some more recent results achieved by some of us [36,37,38,39,40]. Indeed, porphyrins show a very high structural flexibility thanks to the four *meso*, the eight β-pyrrolic, and the two axial positions, which allow a wide variety of chemical functionalizations by playing with the substituents at the periphery of the macrocycle, the nature and the oxidation state of metal center, and the type of axial ligands, for a fine tuning of the optical, electronic, and electrochemical properties in view of an enhanced NLO response.

By using different chemical modifications, several carbonaceous organic moieties such as fullerene, nanotubes, carbon sphere, and graphene [41,42,43,44,45,46,47,48] can efficiently be attached to the porphyrin core to obtain new interesting conjugates [49,50,51,52].

In particular, in recent work [52], some of us investigated, using the Electric Field Induced Second Harmonic generation (EFISH) technique, the second order NLO response in CH_2_Cl_2_ solution of some dyads (3b(Zn) and (6(Zn)-C60) and triads (10b(Zn)-C60) (Figure S1) formed by the Zn(II) complex of *meso*-tetraphenylporphyrin (ZnP) (Figure S2) with an electron donor ferrocene (Fc), and/or an electron acceptor fullerene (C60) moiety connected to the core in 2 or 2,12 β-pyrrolic position via an ethynylphenyl spacer.

In addition to confirming the possibility for ZnP to act both as an electron acceptor or an electron donor moiety (when connected to electron rich Fc or electron deficient C60, respectively), that study surprisingly highlighted for all the investigated compounds negative EFISH responses. Density Functional Theory (DFT) calculations provided an almost null dipole moment (μ) for 3b(Zn) and μ values in the range 3.5–4.8 D for 6(Zn)-C60 and 10b(Zn)-C60, thus suggesting that these compounds feature a low polarity and, as a consequence, a not negligible third-order contribution to their second order NLO response.

**Figure 1:**
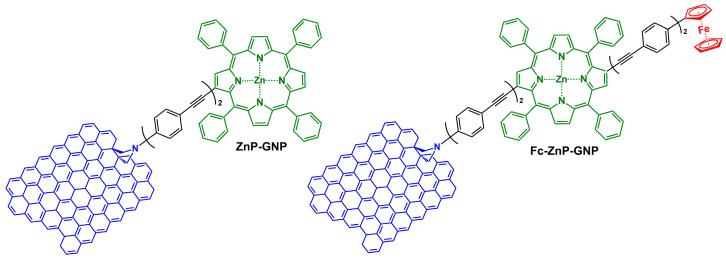
Dyad and triad investigated in this work.

Following these results, this paper represents a part of a larger investigation that we are interested in carrying out on electron donating-accepting multicomponent hybrid systems in search of new promising materials with enhanced nonlinearity. Therefore, in the present research we report and discuss the findings of a combined EFISH and theoretical investigation on the second order NLO properties of some new hybrids, similar to the previous ones, but with two-dimensional graphene nanoplates (GNP) instead of a zero-dimensional C60 moiety, and two ethynylphenyl spacers instead of one (Figure 1). Indeed, the lengthening of the spacer between the ZnP unit and the carbonaceous moiety, and the more π-delocalization of the latter should assure a higher response in comparison to the already studied systems.

GNP can be described as few layers of graphene, produced in high yields in research-based laboratories. Graphene has been a subject of several investigations by the scientific community, because of its unique physical and chemical properties. However, its application has been hindered by its poor solubility due to the high π–π interlayer attraction energies. This problem can be overcome by the functionalization on the sheet surface by either a covalent or noncovalent method which opens new directions to introduce various organic molecules into graphene sheets [53]. As reported, the π-delocalized electron system of graphene acts as an electron acceptor when connected to a metal porphyrin [54]. Therefore, in our GNP-containing hybrids, push–pull interaction can be envisaged, that may be exploited to boost the second order optical nonlinearity.

## 2. Materials and Methods

### 2.1. Materials

The synthesis of the dyad and triad here investigated involves a multi-step process (see Section 3.1). We prepared GNP as reported in [44]. Then, we prepared and characterized Fc-ZnP-CHO, ZnP-GNP, and Fc-ZnP-GNP as reported in [53]. the parent compounds Br-P, Br_2_-P, EP_2_-CHO, Br-P-CHO, and Fc-P-CHO were also prepared as reported [51,55,56].

We characterized GNP by Scanning Electron Microscopy (SEM) analysis, carried out with a TESCAN VegaII at 10 KV of voltage acceleration with a working distance of 14.67 mm. (Tescan, Brno, Czech Republic).

We recorded the UV–Vis electronic absorption spectra of ZnP-GNP and Fc-ZnP-GNP in CH_2_Cl_2_ solution at room temperature on a Shimadzu UV 3600 spectrophotometer (Shimadzu Corporation, Kyoto, Japan).

### 2.2. EFISH Measurements

For the EFISH experiments, we used freshly prepared 10^−3^ M solutions in CH_2_Cl_2_. We chose a 1.907 μm laser incident wavelength since its second harmonic (at 953 nm) is far enough from the absorption bands of the chromophores in CH_2_Cl_2_ (see Section 3.3), that is a necessary requirement to avoid any resonance effect on the second order NLO response. We obtained the incident 1.907 µm wavelength by Raman shifting the 1.064 µm emission of a Q-switched Nd:YAG laser in a high pressure hydrogen cell (60 bar). The Maker fringe pattern, typical of the EFISH signal, was obtained through a liquid cell with thick windows in the wedge configuration. In the experiment, we synchronized the incident beam with a DC field applied to the solution, with 60 and 20 ns pulse duration, respectively, in order to break its centrosymmetry. We assumed the NLO response to be real because we neglected the imaginary part, and derived it from the experimental γ_EFISH_ value (Equation (1)):

(1) $$\gamma_{EFISH}=\frac{\mu \beta_{\lambda }(-2\omega ;\omega ,\omega )}{5kT}+\gamma (-2\omega ;\omega ,\omega ,0)$$

γ_EFISH_ is the sum of a quadratic dipolar orientational contribution μβ_λ_(−2ω; ω, ω)/5 kT, and of a purely electronic cubic contribution γ(−2ω; ω, ω, 0). μ is the molecular ground state dipole moment, and β_λ_ the projection along the dipole moment direction of the vectorial component β_vec_ of the tensorial quadratic hyperpolarizability working with the incident wavelength λ [57,58].

We performed the EFISH experiments at the Department of Chemistry of the University of Milano (Italy), on a prototype apparatus made by SOPRA (Paris, France), recording firstly the second order response of the pure solvent, then the second order response of the chromophore in solution, and finally the second order response of the solvent again. The EFISH values reported in this paper are the average of twelve consecutive measurements performed on the same sample. The uncertainty of the measure is about ±15%, and the experimental EFISH β_1.907_ values are defined according to the “phenomenological” convention [59].

### 2.3. Computational Details

We used the Gaussian16 suite of programs (Revision A.03; Gaussian, Inc.: Wallingford, CT, USA, 2016) to perform DFT calculations. We optimized the molecular geometry with the 6-311G(d) basis set using the PBE0 functional [60,61] in CH_2_Cl_2_, and we adopted the Polarized Continuum Model in its integral equation formalism (IEFPCM) to describe the solvent effect [62]. We modeled graphene as a single layer of 14 condensed benzene rings and we anchored the porphyrin moiety on the center of the graphene-like system in order to minimize boundary effects. We computed the Second Harmonic Generation (SHG) first, β(−2ω; ω, ω), and second, γ(−2ω; ω, ω, 0), hyperpolarizability tensors by the Coupled Perturbed Kohn–Sham (CPKS) approach and by the Finite Field technique, respectively, at the same frequency of the EFISH experiments (1907 nm). We performed hyperpolarizability calculations at LC-BLYP/6-31G(d) level, in agreement with what was suggested by Wergifosse and Champagne in their thorough investigation on electron correlation effects on the first hyperpolarizability of push–pull π-conjugated systems with polyene and polyyne linkers [63]. We selected a pruned (99,590) grid for computation and use of two-electron integrals and their derivatives. From the full tensors β and γ, we derived the scalar quantities β_‖_ and γ_‖_, respectively, to have a meaningful comparison with the experimental data. β_‖_ corresponds to 3/5 times β_λ_, the projection along the dipole moment direction of the vectorial component of the β tensor, that is, β_‖_ = (3/5) ∑_i_(μ_i_β_i_)/μ, where β_i_ = (1/5)∑_j_(β_ijj_ + β_jij_ + β_jji_) [64,65]. Γ_‖_ is related to the tensor components according to the following: γ_‖_ = (1/15) [3(γ_xxxx_ + γ_yyyy_ + γ_zzzz_) + 2(γ_xxyy_ + γ_xxzz_ + γ_yyzz_ + γ_yyxx_ + γ_zzxx_ + γ_zzyy_) + (γ_xyyx_ + γ_xzzx_ + γ_yzzy_ + γ_yxxy_ + γ_zxxz_ + γ_zyyz_)] [64].

## 3. Results and Discussion

### 3.1. Synthesis

**Scheme 1:**
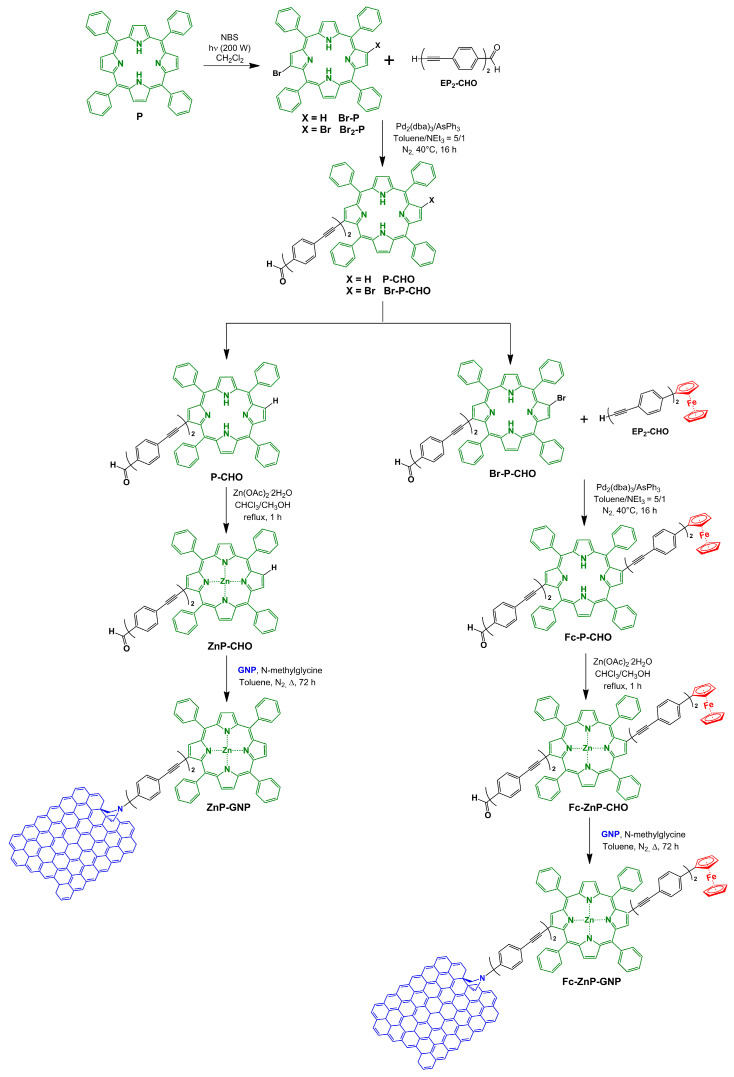
Synthetic route to the investigated compounds.

Although the NLO properties of graphene and porphyrins conjugates have already been investigated, for example by the Z-scan technique [66,67], to the best of our knowledge, the present work is the first EFISH investigation of the second order NLO response of dyad ZnP-GNP and triad Fc-ZnP-GNP. On the other hand, their synthesis has already been reported [53], but since it is not trivial, we wish to recall it (Scheme 1).

We synthesized GNP by a low cost, fast, scalable, and stable fabrication method using a standard 800 W household microwave (MW) oven, developed by some of us [44]. The procedure started with an Asbury Expandable graphite sample (Asbury Carbons, Detroit, MI, USA), where the graphene planes were intercalated with chemical substances such as sulphates and nitrates.

After MW irradiation, the samples were best described by a worm-like morphology with a very large particle area. Using a short ultrasound treatment in isopropyl alcohol, the overall wormlike structures were removed from GNPs. After the treatment, the 2D particles show a lateral dimension of 10 µm and thicknesses < 5 nm, corresponding to several layers of graphene.

Following the GNP synthesis process, we used the new carbon material as the electron acceptor moiety for the formation of dyad ZnP-GNP and triad Fc-ZnP-GNP (Scheme 1).

The synthetic approach that involves the Sonogashira coupling reaction for the formation of carbon–carbon bonds allowed obtaining the desired β-pyrrolic mono and disubstituted compounds.

We chose ethynylphenyl functionalities as molecular bridges, because of their synthetic versatility and their outstanding physicochemical properties. It was previously reported that these linkers assist in a good conduction of the charges due to their high electron density and the extended π-system [65,68,69,70,71].

The first step of the synthesis involved bromination of commercially available free-base porphyrin P with N-bromosuccinimide (NBS) to obtain the monobromo derivative Br-P or the dibromo-porphyrin Br_2_-P. We carried out the regiospecific antipodal bromination of the macrocycle in 2,12 β-pyrrolic position using a light-induced reaction and NBS in CH_2_Cl_2_ [72]. Then, the Sonogashira coupling of Br-P or Br_2_-P with 1.5 equivalents of 4-[(4′-ethynyl)phenyl]-ethynylbenzaldehyde (EP_2_-CHO) afforded derivatives P-CHO and Br-P-CHO in 65 and 36% yield, respectively, after chromatographic purification [56].

For P-CHO, the subsequent step was the insertion of the Zn(II) ion in the cavity of macrocycle, dissolving the free-base in chloroform and adding a 10% excess of a Zn(OAc)_2_ methanol solution, to afford the corresponding complex ZnP-CHO. Finally, the electron acceptor unit GNP was connected to ZnP-CHO by the Prato–Maggini reaction [73], and we obtained dyad ZnP-GNP.

On the other hand, the preparation of triad Fc-ZnP-GNP involved, before complexation of the core with Zn(II) and the Prato–Maggini reaction, a second Sonogashira coupling between Br-P-CHO and two equivalents of EP_2_-Fc, obtaining intermediate Fc-P-CHO in 53% yield [53].

Each intermediate compound of the reaction pathway was characterized and confirmed using mass spectrometry and ^1^H-NMR spectroscopy (Figures S3–S11 in Supplementary Materials). On the other hand, the final products ZnP-GNP and Fc-ZnP-GNP were characterized by X-ray photoelectron spectroscopy (Figures S12 and S13 in Supplementary Materials) and Raman spectroscopy (Figure S14 in Supplementary Materials).

### 3.2. SEM of GNP

**Figure 2:**
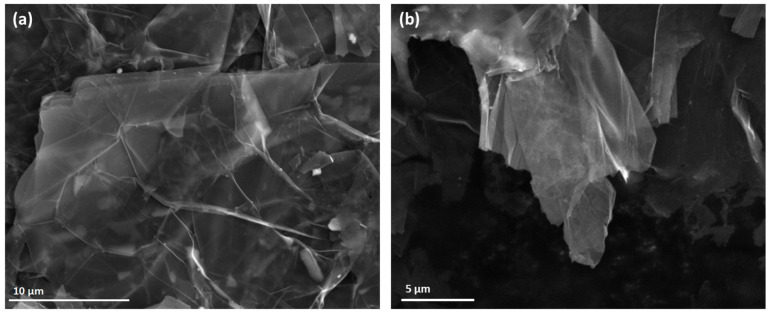
SEM micrographs of GNP sample at different magnifications: (**a**) 10 μm and (**b**) 5 μm.

We characterized the prepared GNP by SEM analysis (Figure 2). The image shows the micrographs of GNP samples obtained after the irradiation power using a standard 800 W household microwave oven. The SEM micrographs show a smooth surface of the pristine GNP and confirm that the intercalated multilayer graphite had undergone expansion with a good dispersion of the material.

The 2D particles show a lateral dimension of around 10 µm, corresponding to several layers of graphene (3–7 layers) [39].

### 3.3. UV–Vis Spectroscopy in Solution

We investigated dyad ZnP-GNP and triad Fc-ZnP-GNP by UV–Vis spectroscopy in CH_2_Cl_2_ solution (that is, in the same solvent used in the EFISH experiments) to exclude any resonance effect on the second order NLO response, and to see if the replacement of C60 with GNP might have an influence on the electronic properties, since the spectra of reference compounds 6(Zn)-C60 and 10b(Zn)-C60 have been previously reported in this solvent [52].

**Figure 3:**
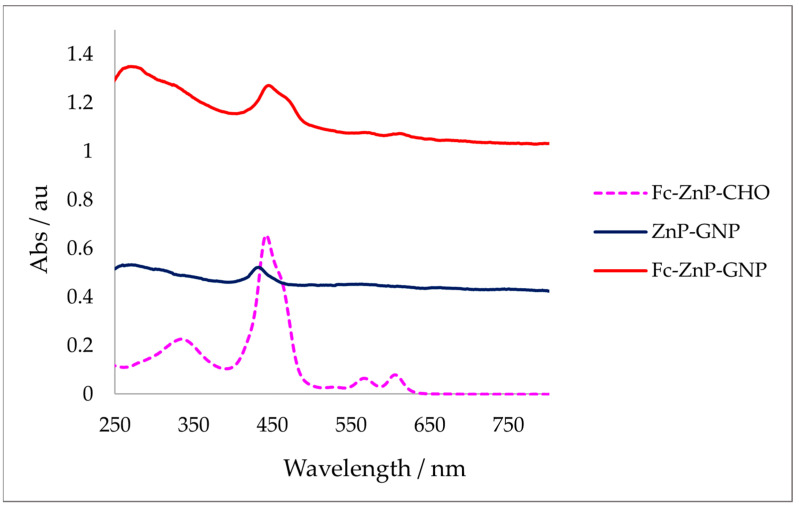
The as-is UV–Vis spectra in CH_2_Cl_2_ of the investigated compounds and of precursor Fc-ZnP-GNP.

Figure 3 shows the as-is spectra, together with that of precursor compound Fc-ZnP-CHO (Scheme 1). A synopsis of the UV–Vis data of the investigated compounds and of reference compounds is reported in Table 1.

The as-is UV–Vis spectra of ZnP-GNP and Fc-ZnP-GNP are dominated by the significant optical density in the near-IR region, due to the plasmonic resonance of GNP [44], that hampers clearly perceiving the typical pattern of porphyrin metal complexes. Indeed, the spectrum of precursor compound Fc-ZnP-CHO, lacking the GNP unit, shows the classical spectroscopic features expected for a metal porphyrin according to the Gouterman’s “four orbital” model [74], that is an intense (ε ≈ 10^5^ M^−1^ cm^−1^) Soret or B band at 400–450 nm, due to a S_0_ → S_2_ transition, and two less intense (ε ≈ 10^4^ M^−1^ cm^−1^) Q bands at 550–650 nm, ascribed to S_0_ → S_1_ transitions. For ZnP-GNP only, the Soret band is evident, while the Q bands disappear in the background, so that their wavelength cannot be safely determined, even normalizing the spectrum (Figure S15). On the other hand, in the spectrum of Fc-ZnP-GNP, both the B band and the two Q bands are noticeable, and the former is accompanied by a shoulder at higher wavelengths, which can be ascribed to the Fc unit, since it is present also in the spectrum of Fc-ZnP-CHO.

The covalent bonding of ZnP to GNP in 2 β-pyrrolic position through two ethynylphenyl spacers causes a 13 nm bathochromic shift of the Soret band, supporting the electron withdrawing character of the carbonaceous unit. The further introduction of a Fc moiety in 12 β-pyrrolic antipodal position as in Fc-ZnP-GNP produces an additional 12 nm red-shift in comparison to ZnP-GNP. Therefore, not only the interaction with GNP perturbs the electronic state of ZnP [46], but this perturbation is also enhanced in the presence of an electron donor moiety in the antipodal position of the core, suggesting an increased π-delocalization within the system and a more evident push–pull character. Indeed, the functionalization of the electron rich β-pyrrolic position with donor substituents was already reported to be an effective way to enhance the second order NLO properties of porphyrins [75]. The effect of the introduction of GNP on the electronic properties of the investigated hybrids is also confirmed by the 3 and 6 nm redshift experienced by the B and Q_β_ bands on going from Fc-ZnP-CHO to Fc-ZnP-GNP.

The comparison of the UV–Vis spectra of ZnP-GNP and Fc-ZnP-GNP with those of 6(Zn)-C60, 10b(Zn)-C60, and ZnP (Figures S16 and S17) allows to evaluate both the effect of the replacement of zero-dimensional C60 with two-dimensional GNP as the acceptor part of the push–pull system, and the effect of the presence of two ethynylphenyl linkers instead of one in β-pyrrolic position of the porphyrin core.

Whereas the introduction on ZnP of a C60 moiety bridged by one ethynylphenyl spacer to the core (ZnP → 6(Zn)C60) leads to significant bathochromic shifts of all the absorption bands (14 nm for the B band and 12 and 9 nm for the Q_α_ abd Q_β_ bands, respectively), the addition of a further ethynylphenyl unit and the replacement of C60 with GNP appears to have basically no effect on the electronic properties, since only a negligible 1 nm hypsochromic shift of the B band is observed comparing 6(Zn)C60 to ZnP-GNP (Figure S14). C60 and GNP appear, thus, to behave as very similar acceptor groups.

Conversely, the linking of a Fc and a C60 unit in antipodal positions of ZnP (ZnP → 10b(Zn)-C60) produces significant red-shifts of all the absorption bands (18, 17, and 14 nm for the B, Q_α_, and Q_β_ bands, respectively), which are further enhanced by adding a second ethynylphenyl spacer between the core and the donor and acceptor groups, and replacing C60 with GNP. Indeed, by comparison of the spectrum of 10b(Zn)-C60 with that of Fc-ZnP-GNP, a 7 nm red-shift of the B band is evident, together with 2 and 8 nm red-shifts for the Q_α_ and Q_β_ bands.

In summary, the UV–Vis spectroscopic investigation would suggest that a GNP unit linked in the 2 β-pyrrolic position of ZnP perturbs the electronic properties of the core in a way that is similar to C60, thus behaving as an acceptor group of comparable strength. The presence of a Fc moiety in 12 β-pyrrolic antipodal position of the macrocycle appears essential to increase the push–pull character of the system, differently from the insertion of a second ethynylphenyl spacer.

### 3.4. EFISH and CP-DFT Investigation of the Second Order NLO Properties

We investigated the second order NLO properties of ZnP-GNP and Fc-ZnP-GNP both experimentally by the EFISH technique and theoretically by DFT (see Section 2.2 and Section 2.3 for the details). We chose to calculate and discuss β_‖_ instead of the spherical averages of the responses (Table S1), since it refers to the projection along the dipole moment direction of the vectorial component of the overall β tensor, which is exactly the figure of merit of the EFISH measure. To better highlight the role of GNP, we also included Fc-ZnP-CHO in our investigation. Figure S18 shows the PBE0/6-311G(d) optimized structures of the three compounds, while Table 2 collects the experimental and theoretical results, together with the data previously reported for the reference compounds with C60 [43].

The experimental γ_EFISH_ and μβ_1907_ values for the GNP-containing hybrids are of the same order of magnitude as those of their C60 counterparts, and, like them, negative [52].

A negative sign of the second order NLO response measured by the EFISH technique can stem from intermolecular interactions or aggregation phenomena that may occur in solution [36,37]. However, as for C60-carrying compounds, as well as ZnP-GNP and Fc-ZnP-GNP, we can safely exclude them, owing to the significant steric hindrance which characterizes A_4_ β-pyrrolic mono or disusbstituted Zn(II) porphyrins [37]. Indeed, the dihedral angles between the aryl rings in the four *meso* positions of the core and the mean plane of the macrocycle are in the range 70°–90° (Figure S16), and induce an overall lowering of the molecular flatness.

For 6(Zn)-C60 and 10b(Zn)-C60, some of us reported the negative sign of γ_EFISH_ to be the result of a not-negligible contribution of the electronic third order cubic term γ_0_(−2ω; ω, ω, 0) to the second order NLO response [52] (see Equation (1)). Indeed, as for other β-pyrrolic mono- and di-substituted Zn(II) porphyrins [39], we observed that in compounds featured by a low polarity, the dipolar orientational contribution μβ_1907_(−2ω; ω, ω)/5 kT to γ_EFISH_ becomes less important and is outstripped by γ_0_(−2ω; ω, ω, 0), whereas in classical push–pull NLO-phores, this latter can be safely overlooked [76].

The new results on GNP-containing conjugates further support these findings. Indeed, experimental γ_EFISH_ and calculated γ_‖_ are almost comparable, at least as order of magnitude, thus confirming the role of third order contributions to the second order NLO response. Moreover, the computed ground state dipole moments (μ) of ZnP-GNP and Fc-ZnP-GNP are very low: 4 and 3.5 times lower than those of reference compounds 6(Zn)-C60 and 10b(Zn)-C60, respectively, and also lower than those of the other β-pyrrolic Zn(II) porphyrins previously studied by some of us [39]. Therefore, from a NLO point of view, we can conclude that GNP behaves as a weaker electron acceptor than C60, and that the dipolar push–pull character of GNP-containing systems decreases in comparison to the C60-containing counterparts, despite the red-shifts of the electronic spectra evidenced by the spectroscopic analysis. Accordingly, the computed β_‖_ values for the GNP-containing compounds are from 1.5 to 4.7 times lower than those of the corresponding C60-substituted complexes. On the other hand, their experimental β_1907_ are apparently higher, but these values must be considered carefully since they are the mathematical result of μβ_1907_/μ and the μ values of GNP-hybrids are very low, as discussed above. As a general rule, indeed, for the systems under investigation the comparison between β_1907_ and β_‖_ is critical, because the former is derived from equation 1 in the assumption of neglecting γ_0_ (−2ω; ω, ω, 0), that, instead, is crucial.

The role of GNP in the decrease of the dipolar character of the compounds under investigation is confirmed by the fair 5.31 D value of μ obtained for Fc-ZnP-CHO, lacking the GNP unit, which is also higher than those of C60-containing systems and thus leads to a higher value of β_‖_. However, even Fc-ZnP-CHO gives rise to a negative γ_EFISH_ value, as expected since the negative μβ_‖_/5 kT value is 53% that of γ_‖_.

Indeed, in an effort to give at least a qualitative idea of the relationship between the theoretical dipolar and cubic components of the second order NLO response of the investigate compounds, we calculated the ratio between μβ_‖_/5 kT and γ_‖_ (Table 2, last column). For ZnP-GNP and Fc-ZnP-GNP, the ratio between the calculated dipolar and cubic contribution decreases significantly, becoming less than 10% and supporting the overwhelming role of γ_0_(−2ω; ω, ω, 0) to γ_EFISH_. On the other hand, the corresponding C60-containing hybrids and Fc-ZnP-CHO show higher ratios, in agreement with their higher μ and β_‖_.

Thus, our combined EFISH and theoretical investigations suggest that the extended π-delocalized structure of GNP enhances the overall polarizability of the GNP-containing hybrids, but without increasing their push–pull character, and therefore leading to chromophores with a not-negligible and significant third order contribution to the second order NLO response.

## 4. Conclusions

In this work, we integrated an experimental EFISH and a theoretical DFT approach to study the second order NLO properties of two hybrids formed by Zn(II) *meso*-tetraphenyl porphyrin (ZnP), electron acceptor graphene nanoplates (GNP), and electron donor ferrocene (Fc).

The UV–Vis absorption spectra suggested that linking a GNP unit in 2 β-pyrrolic position of ZnP perturbs the electronic properties of the porphyrin core, and that this perturbation is similar to that induced by C60. Moreover, the presence of a Fc moiety in antipodal position of the macrocycle appeared essential to increase the push–pull character of the system, differently from the insertion of a second ethynylphenyl spacer.

EFISH measurements produced for the new GNP-conjugates negative γ_EFISH_ and µβ_1907_ values are comparable to those of C60-containing compounds.

Interestingly, the DFT computed ground state μ for GNP-hybrids was very low, even lower than those of the corresponding compounds with C60, supporting for GNP a less electron acceptor character than C60. The peculiar role of GNP in lowering the μ value was sustained by the fair μ of Fc-ZnP-CHO, which matched with its higher ratio between the computed dipolar and cubic contributions (53% vs. less than 10%). Nonetheless, since all the investigated compounds, including Fc-ZnP-CHO, showed a negative second order NLO response, we can conclude that γ_EFISH_ is dominated by the negative pure electronic term γ_0_(−2ω; ω, ω, 0), which overwhelms the dipolar orientational one μβ_1907_(−2ω; ω, ω)/5 kT. The extended π-delocalized structure of GNP is therefore very efficient in increasing the overall polarizability of the hybrids, without increasing their push–pull character, so that they basically behave as third order chromophores.

## Figures and Tables

**Table 1:** Synopsis of the UV–Vis spectroscopic data in CH_2_Cl_2_ of the GNP-containing hybrids in comparison to C60-containing ones and to precursors Fc-ZnP-CHO and ZnP.

Compound	Soret Band λ_max_ (nm)	Q_α_ Band λ_max_ (nm)	Q_β_ Band λ_max_ (nm)
ZnP-GNP	433	nd	nd
Fc-ZnP-GNP	445	567	611
Fc-ZnP-CHO	442	567	605
6(Zn)-C60 ^1^	434	560	598
10b(Zn)-C60	438	565	603
ZnP ^1^	420	548	589

^1^ from ref. [52].

**Table 2:** Synopsis of the experimental EFISH and theoretical DFT results on the investigated compounds in comparison to the reference ones.

Compound	μ (D)	γ_EFISH_ (×10^−36^ esu)	µβ_1907_ (×10^−48^ esu) (β_1907_ × 10^−30^ esu)	β_‖_ (×10^−30^ esu)	μβ_‖_/5 kT (×10^−36^ esu)	γ_‖_ (×10^−36^ esu)	Dipolar vs. Cubic Contribution % ^3^
ZnP-GNP	1.23	−3160	−650 (−528) ^2^	20	120	−1890	6.3
Fc-ZnP-GNP	1.17	−8800	−1880 (−1607) ^2^	9	75	−4388	1.7
Fc-ZnP-CHO	5.31	−4130	−850 (−160) ^2^	94	2920	−5484	53
6(Zn)-C60 ^1^	4.77	−3470	−720 (−151) ^2^	30	696	−1543	45
10b(Zn)-C60 ^1^	4.14	−6410	−1330 (−321) ^2^	42	845	−3225	26

^1^ from ref. [52]. ^2^ given by μβ_1907_/μ. ^3^ given by [(μβ_‖_/5 kT)/γ_‖_] × 100.

## Data Availability

The data presented in this study are available in the article and in the Supplementary Materials.

## Supplementary Materials

The following supporting information can be downloaded at: https://www.mdpi.com/article/10.3390/ma16155427/s1, Figure S1: Dyads and triads already investigated by some of us; Figure S2: Synopsis of chemical structures; Figure S3: FAB spectrum of compound P-CHO using as matrix NBA; Figure S4: ^1^H-NMR in CDCl_3_ of compound P-CHO; Figure S5: ^1^H-NMR in CDCl_3_ of compound ZnP-CHO; Figure S6: FAB spectrum of compound Br-P-CHO using as matrix NBA: Figure S7: ^1^H-NMR in CDCl_3_ of compound Br-P-CHO; Figure S8: MALDI spectrum of compound Fc-P-CHO using as matrix gentisic acid; Figure S9: ^1^H-NMR in CDCl_3_ of compound Fc-P-CHO; Figure S10: FAB spectrum of compound Fc-ZnP-CHO using as matrix NBA; Figure S11: ^1^H NMR in C_6_D_6_ of compound Fc-ZnP-CHO; Figure S12: (a) N 1s and (b) Zn 2p_3/2,1/2_ photoemission regions of ZnP-GNP; Figure S13: (a) Fe 2p_3/2,1/2_ and (b) N 1s photoemission regions of Fc-ZnP-GNP; Figure S14: Raman spectrum using a 600 l/mm grating in the spectral range 200–3000 cm^−1^ of Fc-ZnP-GNP; Figure S15: Comparison of the normalized UV-Vis spectra of Fc-ZnP-CHO, ZnP-GNP and Fc-ZnP-GNP in CH_2_Cl_2_; Figure S16: Comparison of the normalized UV–Vis spectra of ZnP, 6(Zn)-C60 and ZnP-GNP in CH_2_Cl_2_; Figure S17: Comparison of the normalized UV–Vis spectra of ZnP, 10b(Zn)-C60 and Fc-ZnP-GNP in CH_2_Cl_2_; Figure S18: PBE0/6-311G(d) optimized structures of (a) ZnP-GNP, (b) Fc-ZnP-GNP and (c) Fc-ZnP-CHO; Table S1: Calculated spherical averages of the responses for the investigated compounds.
