# Supplementary material for: Nonlinear Optical Properties of Zn(II) Porphyrin, Graphene Nanoplates, and Ferrocene Hybrid Materials
Source: Materials (Basel). 2023 Aug 2;16(15):5427. doi: 10.3390/ma16155427 (PMC10419410; doi:10.3390/ma16155427)
Supplement: Supplementary file 1 [file materials-16-05427-s001.zip › materials-2482392-supplementary.pdf]

Supporting Information

Nonlinear Optical Properties of Zn(II) Porphyrin, Graphene Nanoplates, and Ferrocene Hybrid Materials

Francesca Limosani [1], Francesca Tessore [2,*], Alessandra Forni [3], Angelo Lembo [1], Gabriele Di Carlo [2], Cecilia Albanese [2], Stefano Bellucci [4] and Pietro Tagliatesta [1]

[1] Department of Chemical Science and Technologies, University of Rome "Tor Vergata", Via della Ricerca Scientifica 1, 00133 Rome, Italy; francesca.limosani@uniroma2.it (F.L.); angelo.lembo@uniroma2.it (A.L.); pietro.tagliatesta@uniroma2.it (P.T.)
[2] Department of Chemistry, University of Milan, Via C. Golgi 19, 20133 Milan, Italy; francesca.tessore@unimi.it (F.T.); gabriele.dicarlo@unimi.it (G.D.C.); cecilia.albanese@unimi.it (C.A.)
[3] CNR-SCITEC, Istituto di Scienze e Tecnologie Chimiche "G. Natta", Via Golgi 19, 20133 Milan, Italy; alessandra.forni@scitec.cnr.it
[4] INFN-National Laboratories of Frascati Via Enrico Fermi 54, 00044 Frascati, Italy; bellucci@lnf.infn.it
* Correspondence: francesca.tessore@unimi.it; Tel.: +39-0250314398

Table of Contents

Chemical Structures 3

Experimental 4

Mass spectrometry and ^{1}H-NMR spectroscopy 5

FAB spectrum of P-CHO 5

^{1}H-NMR spectrum of P-CHO 5

Elemental Analysis 6

$C_{37}H_{22}N_4O$ *calc.:* C, 82.51; H, 4.12; N, 10.40; O, 2.97 // *found:* C, 82.48; H, 4.13; N, 10.43; O, 2.96. . 6

^{1}H-NMR spectrum of ZnP-CHO 6

Elemental Analysis 6

$C_{37}H_{20}N_4OZn$ *calc.:* C, 73.82; H, 3.35; N, 9.31; O, 2.66; Zn, 10.86 // *found:* C, 73.89; H, 3.34; N, 9.28; O, 2.67; Zn, 10.82. 6

FAB spectrum of Br-P-CHO 7

^{1}H-NMR spectrum of Br-P-CHO 7

Elemental Analysis 7

$C_{37}H_{21}BrN_4O$ *calc.:* C, 71.97; H, 3.43; Br, 12.94; N, 9.07; O, 2.59 // *found:* C, 71.86; H, 3.44; Br, 12.99; N, 9.11; O, 2.60 7

MALDI spectrum of Fc-P-CHO 8

^{1}H-NMR spectrum of Fc-P-CHO 8

Elemental Analysis 8

FAB spectrum of Fc-ZnP-CHO 9

^{1}H-NMR spectrum of Fc-ZnP-CHO 9

Elemental Analysis 9

X-Ray Photoelectron spectroscopy 10

Compound ZnP-GNP 10

Compound Fc-ZnP-GNP 11

Raman spectroscopy 12

Compound Fc-ZnP-GNP 12

UV-Vis spectroscopy 12

DFT theoretical calculations 14

Chemical Structures

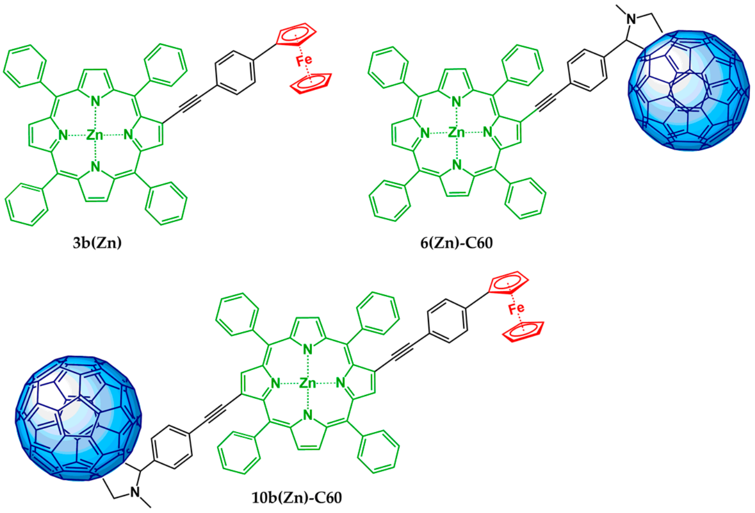

Figure S1. Dyads and triads already investigated by some of us

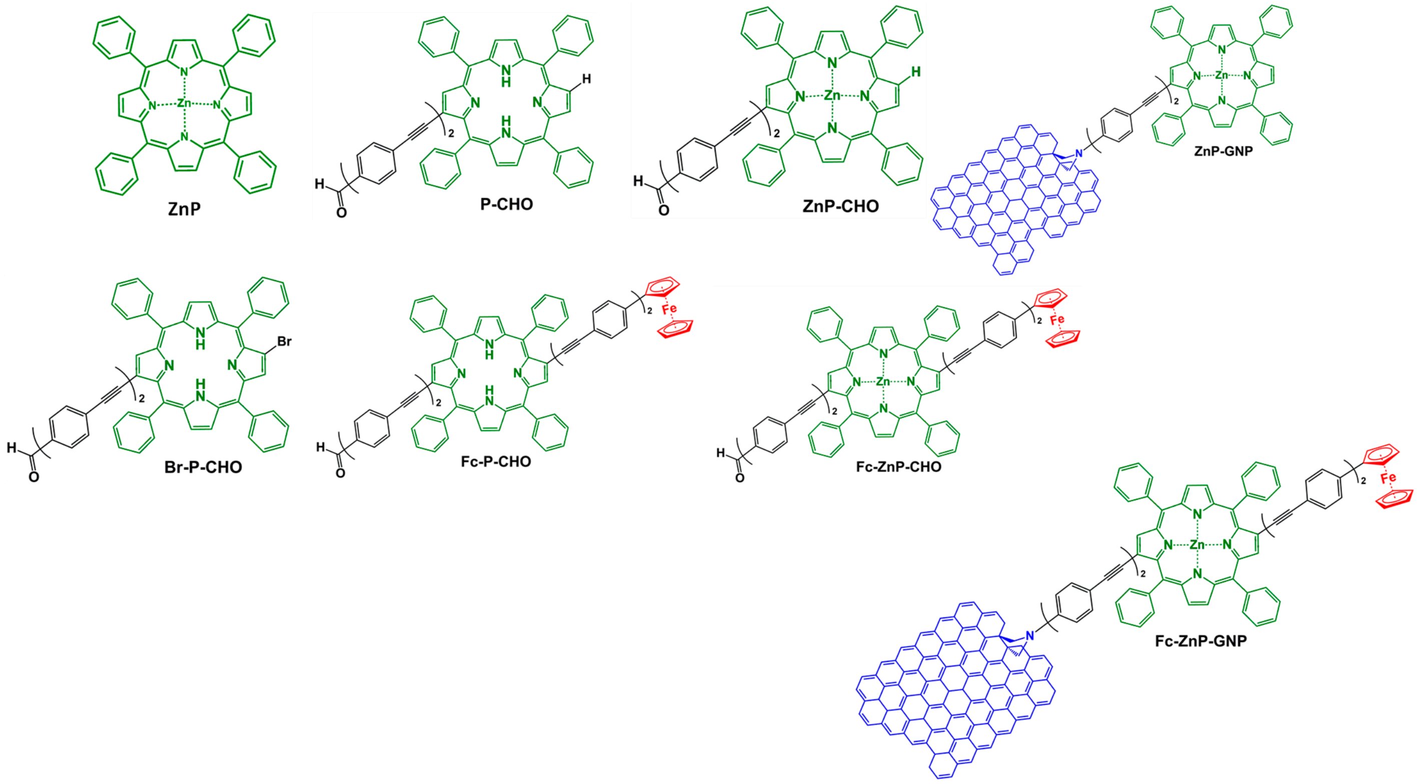

Figure S2. Synopsis of chemical structures

Experimental

General Methods. ^{1}H-NMR spectra were recorded as $CDCl_3$ and C_6D_6 solutions on a Bruker AM-300 instrument using residual solvent signal as an internal standard. Chemical shifts are given as δ values. FAB mass spectra were measured on a VG-4 spectrometer using m-nitrobenzyl alcohol (NBA) as a matrix. Matrix-assisted laser desorption/ionization time of flight (MALDI-TOF) mass spectra were performed with a MALDI-TOF Reflex IV instrument (Bruker-Daltonics) in reflector mode, using a 337 nm nitrogen laser (8 Hz). A 2 mg/mL 2,5-dihydroxybenzoic acid (gentisic acid) solution in CH_3CN/TFA (0.1% solution) was used as a matrix. Electronic absorption spectra were recorded in CH_2Cl_2 solution at room temperature on a Shimadzu UV 3600 spectrophotometer (Shimadzu Corporation, Kyoto, Japan).

Raman spectra were obtaining by using a confocal Raman microscope Invia Renishaw, endowed with a 633 nm laser, a RenCam CCD detector, 1024x256 pixels (200-1060 nm), an encoded xyz stage (replacement precision: 100nm), using an 1800 l/mm grating.

XPS measurements were obtained from a modified Omicron NanoTechnology MXPS system. The spectra were excited by achromatic Mg $K\alpha$ and Al $K\alpha$ photons (hν = 1253.6 and 1486.6 eV, respectively), generated operating the anode at 14-15 kV, 10-20 mA. All the samples were mounted on metal tips as thin layers of pressed powders. Experimental spectra were theoretically reconstructed by fitting the peaks to symmetric pseudo-Voigt functions (linear combination of gaussian and lorentzian peaks) and the background to a Shirley or a linear function. XPS atomic ratios (±10% associated error) between relevant core lines were estimated from experimentally determined area ratios corrected for the corresponding theoretical cross sections and for a square root dependence of the photoelectron's kinetic energies. All binding energies were referenced to the lowest lying C 1s peak component, taken at 285.0 eV, which was assumed to be related to the aromatic C atoms

Elemental analyses were carried out with a PerkinElmer CHN 2400 instrument in the Analytical Laboratories of the Department of Chemistry at the University of Milan.

Mass spectrometry and ^{1}H-NMR spectroscopy

FAB spectrum of P-CHO

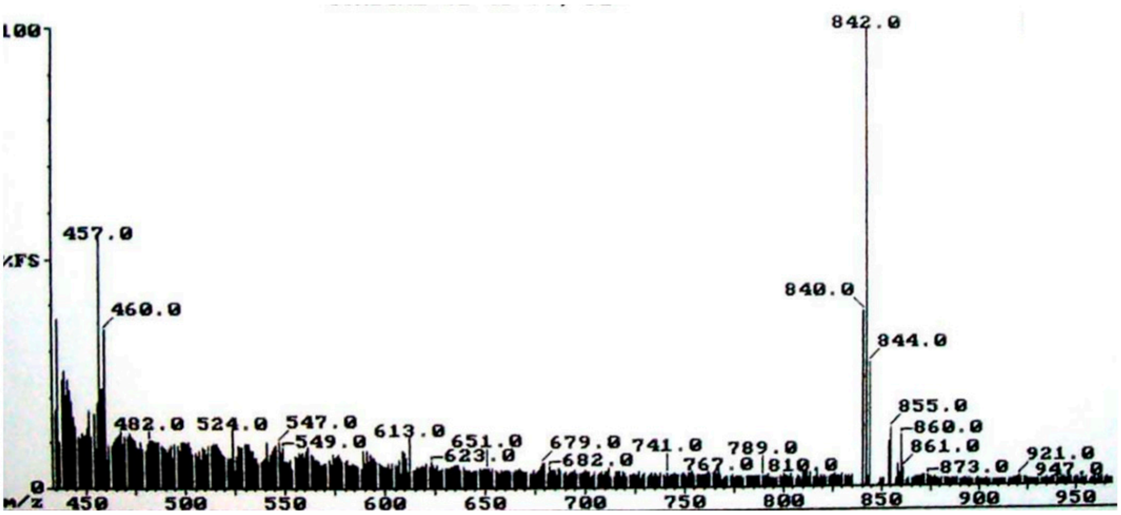

Figure S3. FAB spectrum of compound **P-CHO** using as matrix NBA

^{1}H-NMR spectrum of P-CHO

^{1}H-NMR (300 MHz, $CDCl_3$): δ (ppm) 10.06 (s, 1H), 9.10 (s,1H), 8.90 (s, 2H), 8.85 (d, 3*J*) 5.9 Hz, 1H), 8.79 (s, 2H), 8.77 (d, 3*J*) 5.9 Hz, 1H), 8.23 (br m, 5H), 7.91 (d, 3*J*) 7.8 Hz,2H), 7.80-7.66 (br m, 17H), 7.54 (d, 3*J*) 7.8 Hz, 2H), 7.38 (d, 2H; *J*) 7.8), -2.65 (s, 2H).

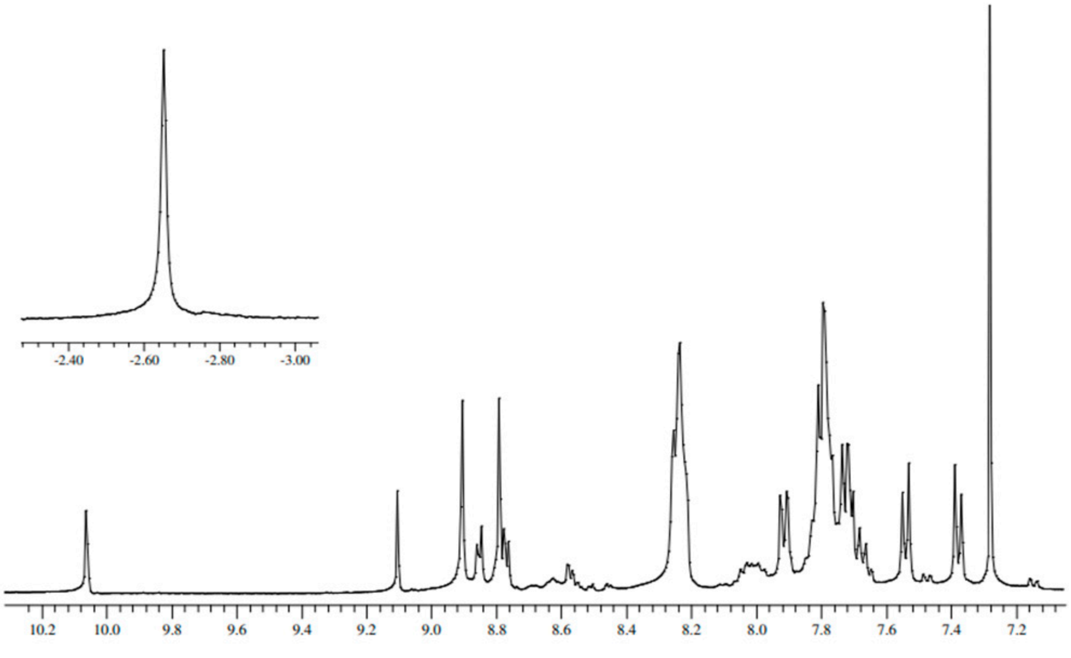

Figure S4. ^{1}H-NMR in $CDCl_3$ of compound **P-CHO**

Elemental Analysis

$C_{37}H_{22}N_4O$ *calc.:* C, 82.51; H, 4.12; N, 10.40; O, 2.97 // *found:* C, 82.48; H, 4.13; N, 10.43; O, 2.96.

^{1}H-NMR spectrum of ZnP-CHO

^{1}H-NMR (300 MHz, $CDCl_3$): δ (ppm) 10.04 (s, 1H), 9.26 (s, 1H), 8.95 (s, 2H), 8.93 (s, 2H), 8.90 (d, 3*J*) 5.3 Hz, 1H), 8.79 (d, 3*J*) 5.3 Hz, 1H), 8.23 (br m, 10H), 7.90 (d, 3*J*) 7.6 Hz, 2H), 7.78 (b m, 10H), 7.69 (d, 3*J*) 6.0 Hz, 2H), 7.55 (d, 3*J*) 8.3 Hz, 2H), 7.40 (d, 3*J*) 8.3 Hz, 2H).

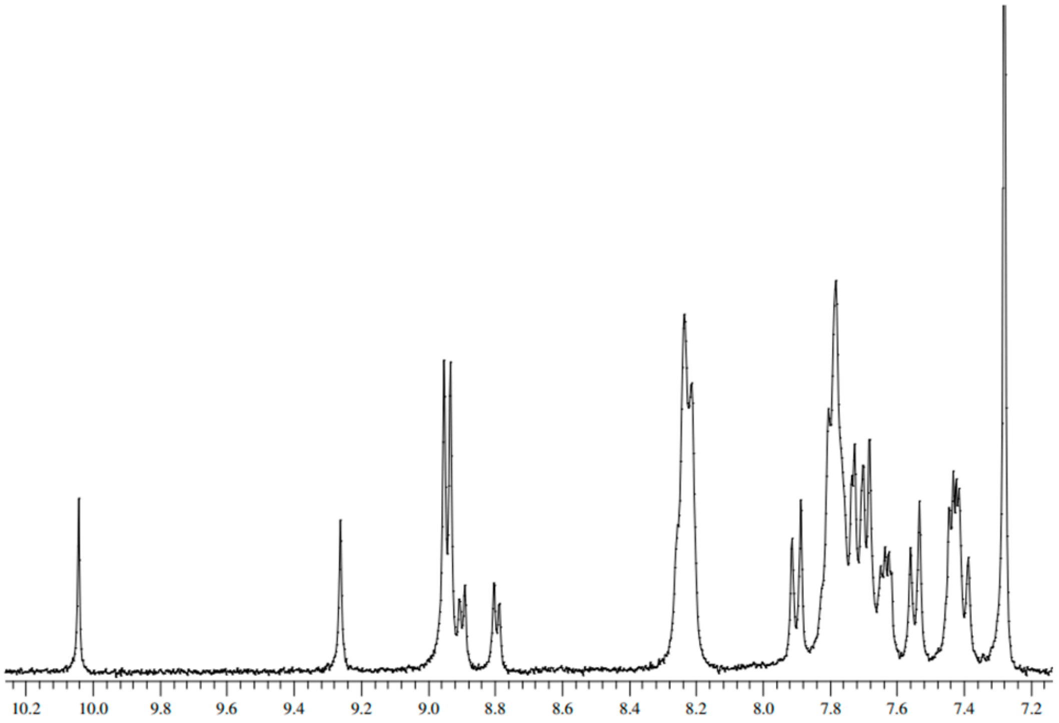

Figure S5. ^{1}H-NMR in $CDCl_3$ of compound **ZnP-CHO**

Elemental Analysis

$C_{37}H_{20}N_4OZn$ *calc.:* C, 73.82; H, 3.35; N, 9.31; O, 2.66; Zn, 10.86 // *found:* C, 73.89; H, 3.34; N, 9.28; O, 2.67; Zn, 10.82.

FAB spectrum of Br-P-CHO

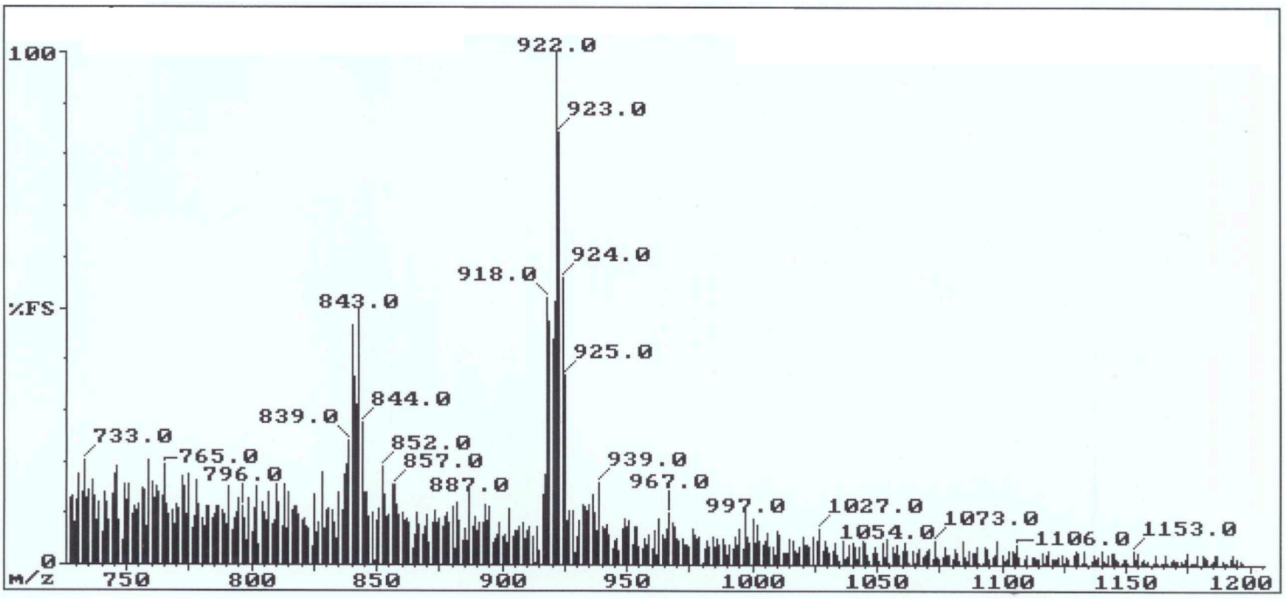

Figure S6. FAB spectrum of compound **Br-P-CHO** using as matrix NBA

^{1}H-NMR spectrum of Br-P-CHO

^{1}H-NMR (300 MHz; $CDCl_3$): δ(ppm) 10.06 (s, 1H), 9.11 (s, 1H), 8.90-7.60 (br m, several signals difficult to resolve and integrate, 25H), 7.54 (d, 2H, J = 9.1 Hz), 7.48 (d, 2H, J= 7.2 Hz), 7.37 (d, 2H, J=7.2 Hz), 7.12 (d, 1H, J = 11.1 Hz), -2.65 (s, 2H); UV-vis (Toluene): λ_{max} (log ε) 432 (5.3), 526 (4.8), 564 (4.6), 604 (4.5), 662 (4.3)

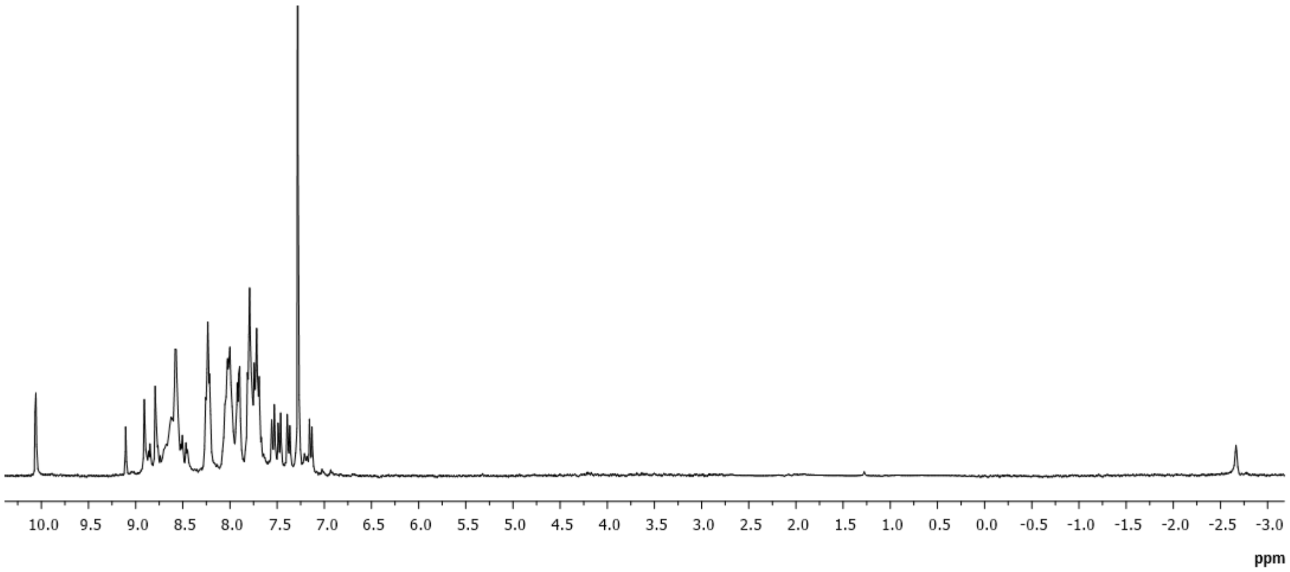

Figure S7. ^{1}H-NMR in $CDCl_3$ of compound **Br-P-CHO**

Elemental Analysis

$C_{37}H_{21}BrN_4O$ *calc.:* C, 71.97; H, 3.43; Br, 12.94; N, 9.07; O, 2.59 // *found:* C, 71.86; H, 3.44; Br, 12.99; N, 9.11; O, 2.60

MALDI spectrum of Fc-P-CHO

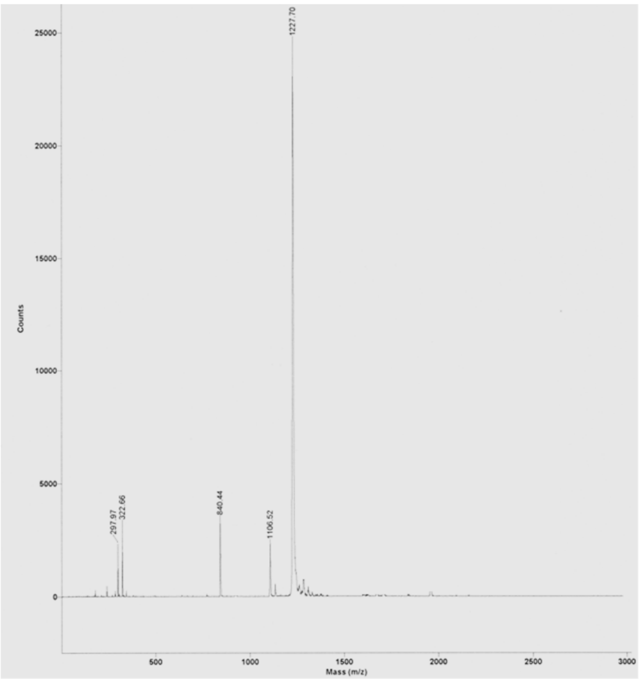

Figure S8. MALDI spectrum of compound **Fc-P-CHO** using as matrix gentisic acid

^{1}H-NMR spectrum of Fc-P-CHO

^{1}H-NMR (400 MHz, C_6D_6): δ (ppm) 9.55 (s, 1H), 9.41 (s, 2H), 8.91-8.65 (m, 5H), 8.16 (s, 4H), 8.05 (s, 5H), 7.62-7.32 (br m, several signals difficult to resolve and integrate, 26H), 4.45 (s, 2H), 4.14 (s, 2H), 3.86 (s, 5H), -2.13 (s, 2H).

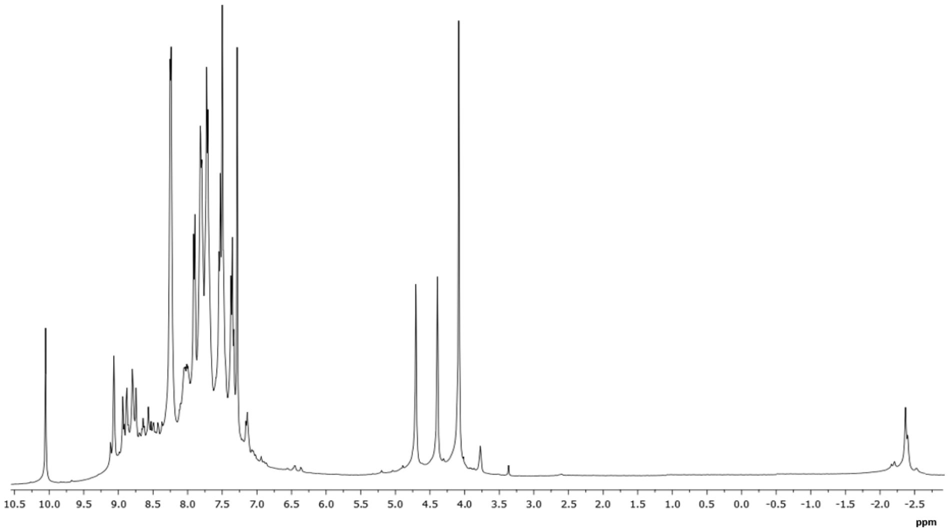

Figure S9. ^{1}H-NMR in $CDCl_3$ of compound **Fc-P-CHO**

Elemental Analysis

$C_{63}H_{38}FeN_4O$ *calc.:* C, 81.99; H, 4.15; Fe, 6.05; N, 6.07; O, 1.73 // *found:* C, 82.02; H, 4.16; Fe, 6.03; N, 6.05; O, 1.74

FAB spectrum of Fc-ZnP-CHO

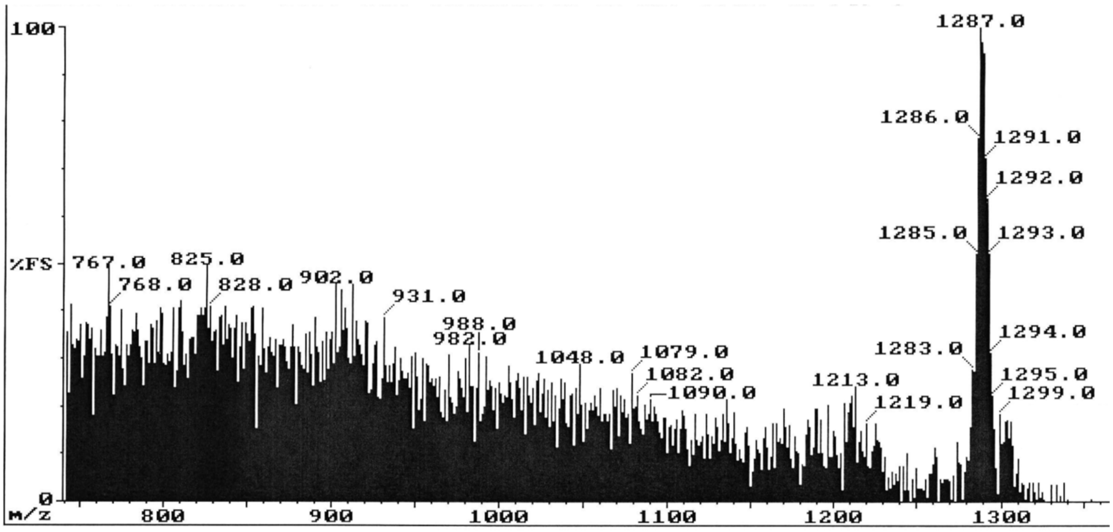

Figure S10. FAB spectrum of compound **Fc-ZnP-CHO** using as matrix NBA

^{1}H-NMR spectrum of Fc-ZnP-CHO

^{1}H-NMR (400 MHz, C_6D_6): δ (ppm) 9.57 (s, 1H), 9.56 (s, 1H), 9.54 (s, 1H), 8.95-8.88 (m, 4H), 8.84 (s, 2H), 8.32-8.26 (m, 6H), 8.24-8.18 (m, 8H), 7.61-7.45 (m, 12H), 7.41-7.30 (m, 8H), 4.47 (s, 2H), 4.14 (s, 2H), 3.91 (s, 5H)

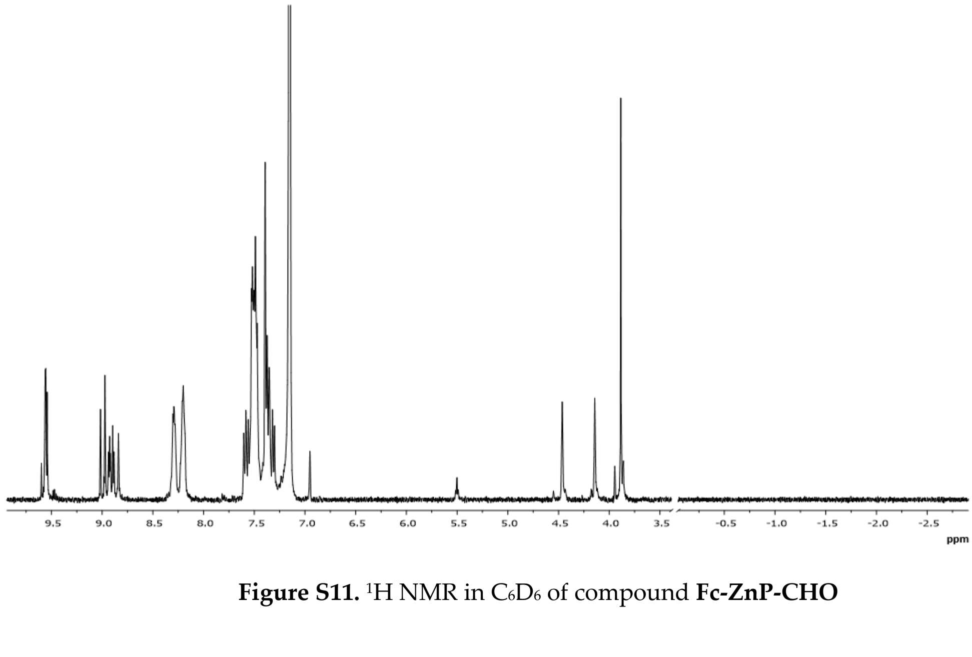

Figure S11. ^{1}H NMR in C_6D_6 of compound **Fc-ZnP-CHO**

Elemental Analysis

$C_{63}H_{36}FeN_4OZn$ *calc.:* C, 76.73; H, 3.68; Fe, 5.66; N, 5.68; O, 1.62; Zn, 6.63 // *found:* C, 76.76; H, 3.67; Fe, 5.64; N, 5.70; O, 1.62; Zn, 6.61

X-Ray Photoelectron spectroscopy

Compound ZnP-GNP

In Figures S10a and S10b the N 1s and Zn $2p_{3/2,1/2}$ photoemission regions for **ZnP-GNP** are reported (Fe is not present in the compound).

The two N 1s distinct components shown in Figure S10a confirm the functionalization of the Zinc 2-[(4'-formyl)phenyl]ethynyl-5,10,15,20-tetraphenylporphyrin on graphene surface. The first peak was attributed to porphyrin nitrogens (399.4 eV) and the second to substituted amine group (402.4 eV) with a 4.0 atomic ratio of the first to the second component. In Figure S10b the Zn $2p_{3/2,1/2}$ component shows a peak at 1022.4 eV, confirming the insertion of the zinc in the macrocycle.

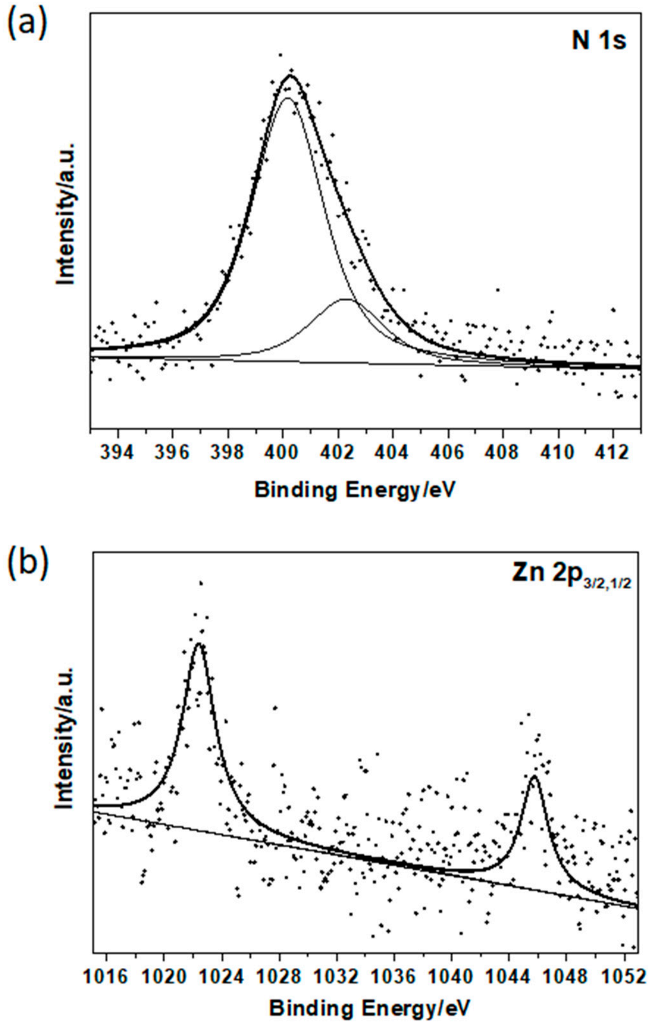

Figure S12. (a) N 1s and (b) Zn $2p_{3/2,1/2}$ photoemission regions of **ZnP-GNP**.
Experimental curve (dots) and peak-fit components (lines) are shown together

Compound Fc-ZnP-GNP

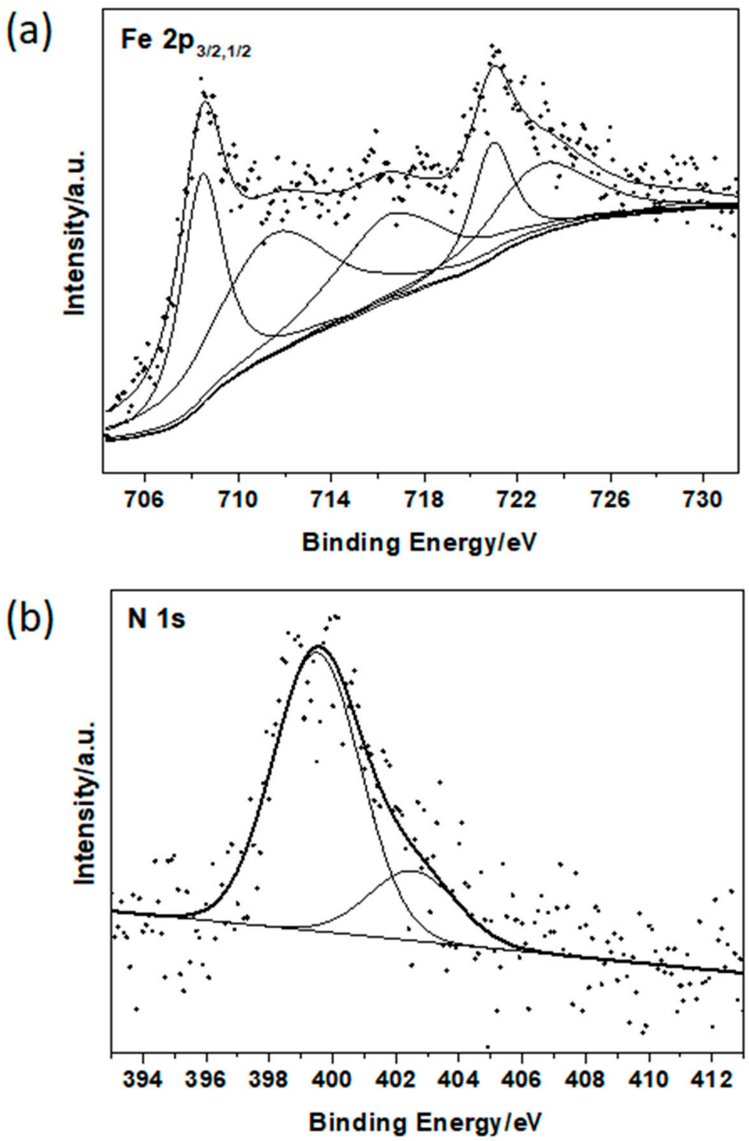

Figure S13. (a) Fe 2p$_{3/2,1/2}$ and (b) N 1s photoemission regions of **Fc-ZnP-GNP**. Experimental curve (dots) and peak-fit components (lines) are shown together.

Raman spectroscopy

Compound Fc-ZnP-GNP

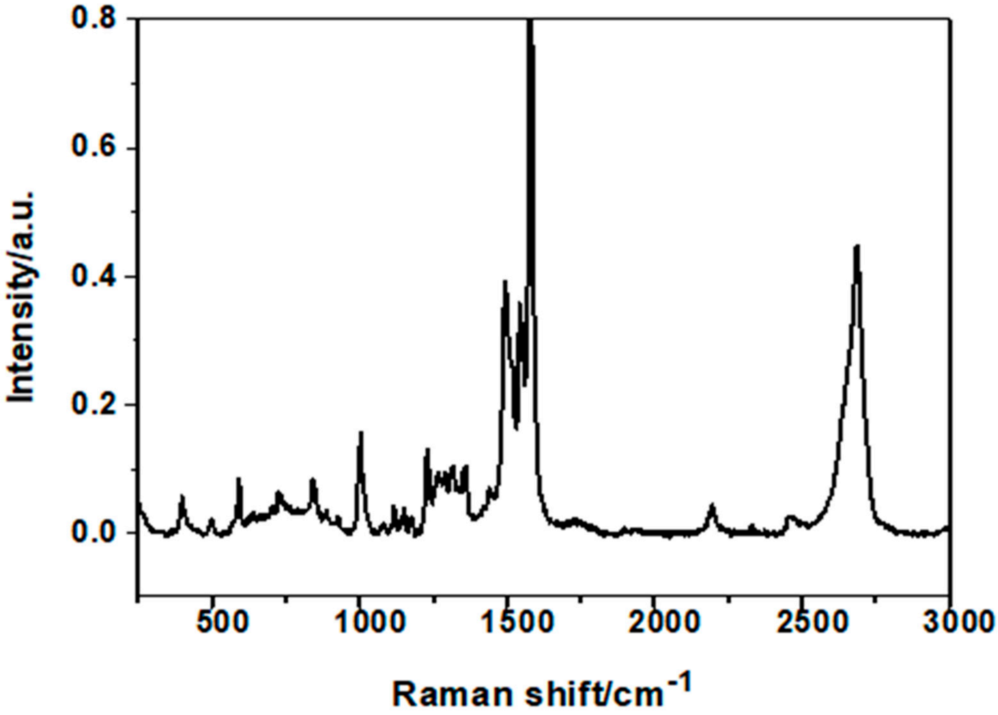

Figure S14. Raman spectrum using a 600 l/mm grating in the spectral range 200 - 3000 cm^{-1} of **Fc-ZnP-GNP**

UV-Vis spectroscopy

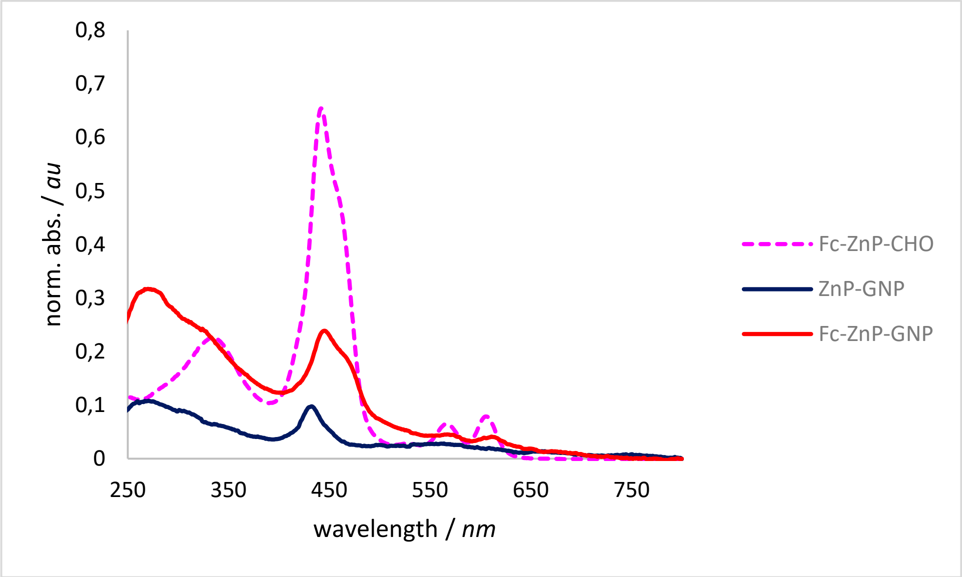

Figure S15. Comparison of the normalized UV-Vis spectra of Fc-ZnP-CHO, ZnP-GNP and Fc-ZnP-GNP in CH_2Cl_2

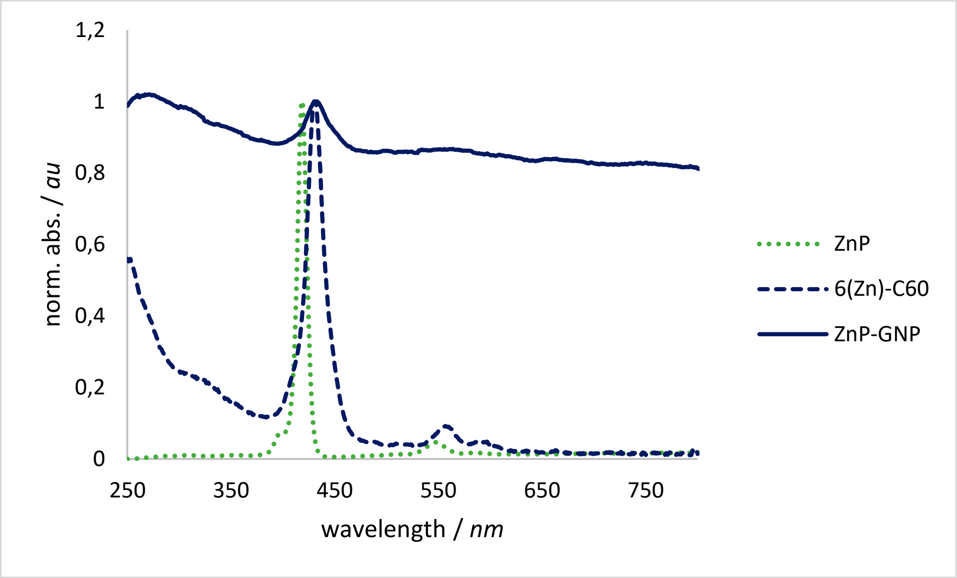

Figure S16. Comparison of the normalized UV-Vis spectra of ZnP, 6(Zn)-C60 and ZnP-GNP in CH_2Cl_2

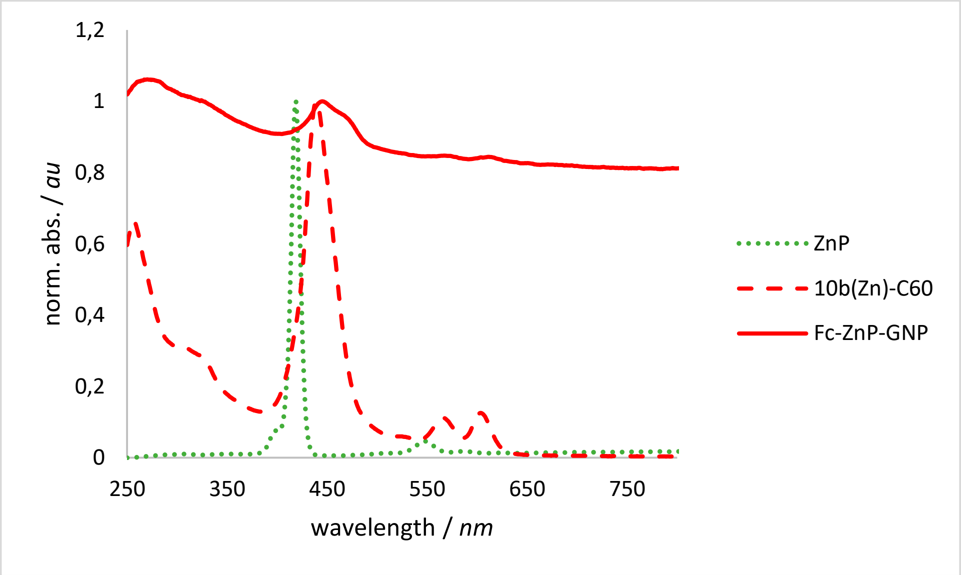

Figure S17. Comparison of the normalized UV-Vis spectra of ZnP, 10b(Zn)-C60 and Fc-ZnP-GNP in CH_2Cl_2

DFT theoretical calculations

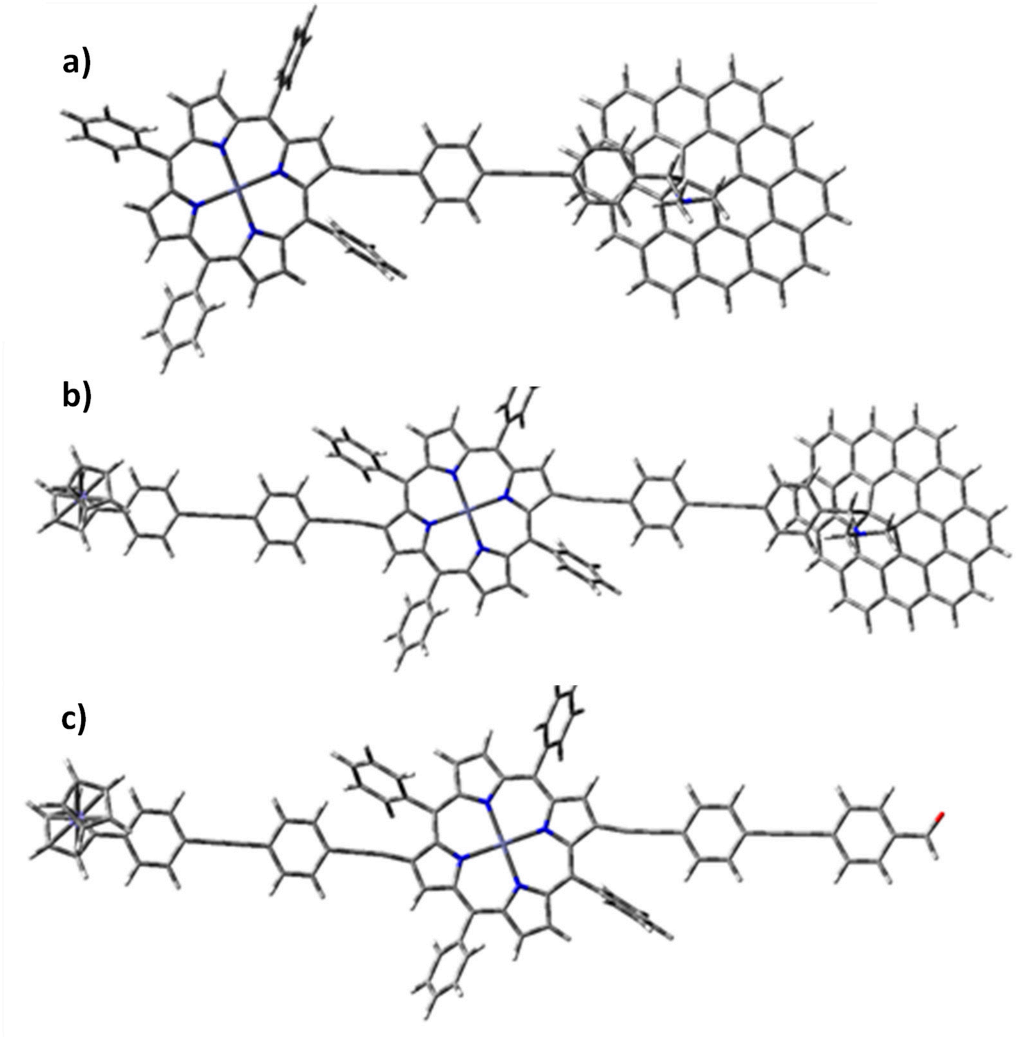

Figure S18. PBE0/6-311G(d) optimized structures of a) ZnP-GNP, b) Fc-ZnP-GNP and c) Fc-ZnP-CHO.

Table S1. Calculated spherical averages of the responses for the investigated compounds

Compound	μ (D)	$<\beta_{\|\|}>$ (x 10^{-30} esu)	$\mu<\beta_{\|\|}>$ /5kT (x 10^{-36} esu)	$\gamma_{\|\|}$ (x 10^{-36} esu)	% of dipolar *vs* cubic contribution
**ZnP-GNP**	1.23	26	155	-1890	8.2
**Fc-ZnP-GNP**	1.17	20	114	-4388	2.6
**Fc-ZnP-CHO**	5.31	103	2659	-5484	48
